# NoRCa: a register-based data source for cancer research in northern Sweden

**DOI:** 10.1186/s12885-026-16559-1

**Published:** 2026-07-17

**Authors:** Cecilia Hultstrand, David Olsson, Senada Hajdarevic, Ove Andrén, Ove Björ

**Affiliations:** 1 Regional Cancer Centre North. Norrlands universitetssjukhus, Umeå, 901 85 Sweden; 2 Department of Nursing, Umeå University, Umeå, 901 87 Sweden; 3 Department of Public Health and Clinical Medicine, Family Medicine, Umeå University, Umeå, 901 87 Sweden; 4 Department of Diagnostics and Intervention, Umeå University, Umeå, 901 87 Sweden

**Keywords:** Cancer, Register data, Database, Inequity, Rural health, Early detection

## Abstract

**Background:**

Rural populations often have poorer health outcomes than urban residents due to limited healthcare access, long travel distances, and diagnostic delays. Northern Sweden, covering 55% of the country but only 10% of its population, exemplifies these challenges. Yet research on cancer care and outcomes in the region remains scarce. To better understand structural factors underlying these inequities, we developed the Northern Region Cancer (NoRCa) database. The aim of this article is to describe the database, outline its coverage and linkage structure, and discuss how leveraging this resource has the potential to promote rural cancer research.

**Methods:**

NoRCa includes all individuals diagnosed with cancer in the Northern Healthcare Region between 2007 and 2022. Using the Swedish personal identification number, each case was linked to national registers containing data on cancer type and stage, inpatient and specialist outpatient care, prescribed medications, cause of death, demographics, and socioeconomic factors. Geographic information includes residential location and distance to the nearest primary care centre and hospital at diagnosis.

**Results:**

The database contains 88,796 incident cancer cases. By integrating clinical, socioeconomic, and geographic data, NoRCa offers a foundation for advancing rural cancer research and informing efforts toward more equitable cancer care.

**Conclusions:**

The NoRCa database is a unique register-based data source which has the potential to be used in numerous epidemiological studies, offering several possible research directions. Moreover, the insights generated through our research agenda can inform improvements in the healthcare system, supporting earlier detection efforts and promoting better health outcomes for the population.

## Background

Previous research indicates that rural residents tend to have poorer health outcomes compared with urban residents [1, 2]. Rurality is increasingly recognised as a social determinant of health, reflecting structural conditions that may influence health beyond factors at individual level. People living in rural areas often face challenges related to healthcare access, including limited availability of healthcare services, shortages of healthcare professionals, and long travel distances to care [3].

These structural barriers are further compounded by the scarcity of research dedicated to rural health issues [4, 5]. Moreover, rural populations are underrepresented in clinical trials and biomedical research [6, 7], potentially contributing to less contextually adapted healthcare.

### Northern Sweden

**Fig. 1:**
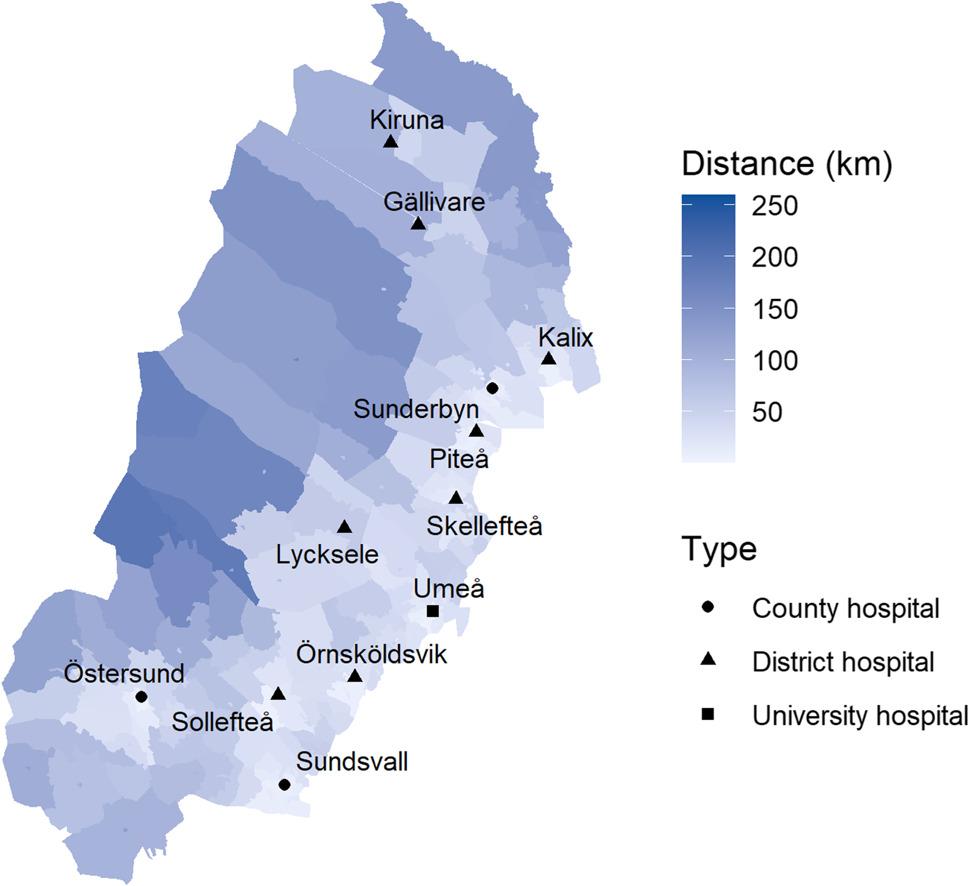
Map of the study area with average distance to nearest hospital per DeSO area

Northern Sweden is geographically vast and sparsely populated, reflecting many of the challenges described above. The Northern Healthcare Region (NHCR) consists of four counties: Västernorrland, Jämtland Härjedalen, Västerbotten, and Norrbotten. The population of the NHCR was 901,407 in 2022. This corresponds to approximately 10% of Sweden’s total population, while covering 55% of Sweden’s land area, highlighting the pronounced geographic dispersion of the population and its implications for healthcare access. Figure 1 provides an overview of the study area with average distances to nearest hospital per Demographic Statistical Areas (DeSO).

Healthcare in Sweden is tax-financed and decentralized, with the regions responsible for the provision of most healthcare services. First line of healthcare in the NHCR is primary care, provided by 135 primary healthcare centres. The second line of healthcare is provided by one university hospital, four county hospitals (Länssjukhus), and seven district hospitals (Länsdelssjukhus), of which one is set to be closed down in the near future. University hospitals provide highly specialized care. County hospitals have the competence and medical equipment to cover most areas of disease. District hospitals, on the other hand, are more limited and therefore cannot perform all types of specialized care [8].

### Cancer and cancer care

In Sweden, the cancer incidence is continuously increasing [9], reflecting global epidemiological trends [10, 11]. Together with cardiovascular diseases, cancer is the leading cause of death in Sweden [12]. To mitigate this growing public health challenge, improving survival and minimizing suffering caused by cancer are imperative [13].

Evidence shows that diagnosing cancer at an earlier stage substantially improves patient outcomes [14]. The importance of early detection, in combination with timely and accessible treatment, is well established in strengthening cancer control [15–17]. However, despite the overarching goal of Swedish healthcare services— to ensure good health and care on equal terms for the entire population [18]—cancer care has been described as unevenly distributed across the country. Significant regional disparities persist, including inequities in access to care, treatment availability, and timeliness of cancer detection [19]. These differences may reflect structural variations in healthcare access, such as service availability, geographic distance, and waiting times [19]. For early detection, disparities may also be linked to differences in capacity and resources of primary healthcare to identify alarm symptoms and early signs of cancer [19].

Swedish data demonstrate regional variation in stage at diagnosis for prostate, breast, rectal, and colon cancer. For example, the proportion of patients diagnosed with stage IV rectal cancer is 9% points higher in Västerbotten than in Norrbotten. Similarly, for lung cancer, the proportion of stage IV diagnoses is 11, 11, 10, and 17% points higher in Norrbotten, Västerbotten, Västernorrland, and Jämtland Härjedalen, respectively, compared with the region in Sweden with the lowest proportion [19]. Furthermore, compared with Sweden overall, the population in northern Sweden demonstrates lower cancer survival among women, while survival among men is similar [20].

Adding to the complexity, factors beyond rurality and healthcare accessibility, such as socioeconomic inequities, also influence early cancer detection and survival [21, 22]. The Swedish National Board of Health and Welfare has reported higher cancer mortality in areas characterized by more disadvantaged socioeconomic conditions [23]. Globally, persistent inequities in cancer outcomes highlight the need for continuous monitoring to ensure adequate prevention and care for people [24]. Improving cancer outcomes requires a comprehensive view of the healthcare system, as cancer care does not work in isolation from the rest of the system [25]. Thus, access to data that enables monitoring and exploration of processes of care seeking and diagnostic delay across the entire healthcare system, including primary care, could improve the diagnostic process, prevention, and timely detection of cancer.

In summary, cancer remains a major public health challenge, and its incidence continues to rise. At the same time, research focusing on healthcare in rural and sparsely populated areas is scarce. This lack of research is concerning, as individuals living in these regions have a lower likelihood of cancer-specific and all-cause survival [26]. To better understand the healthcare structures contributing to these inequities, we developed the Northern Region Cancer (NoRCa) database.

## Methods

### Aim

The aim of this article is to describe the database, outline its coverage and linkage structure, and discuss how leveraging this resource has the potential to promote rural cancer research.

### The NoRCa database

**Fig. 2:**
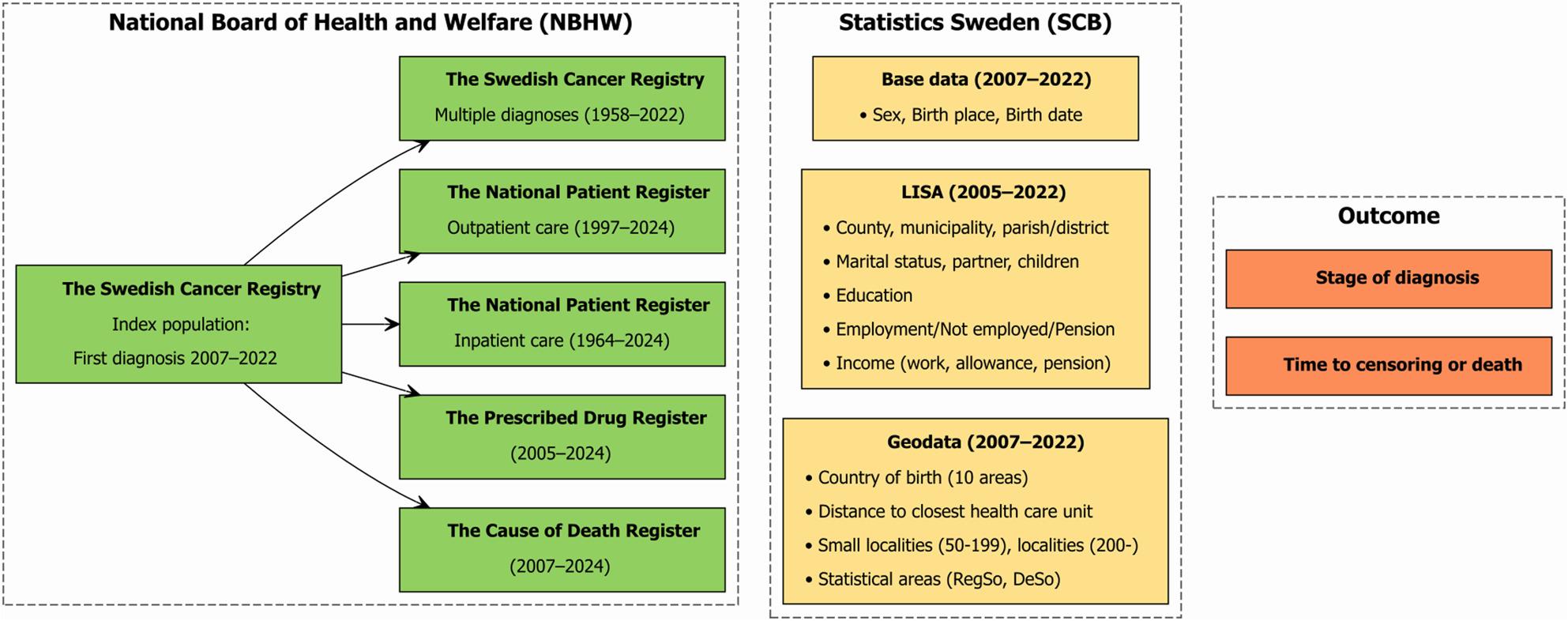
Flowchart of data linkages and sources. Index patients were identified in the Swedish Cancer Registry based on their first recorded diagnosis between January 1, 2007, and December 31, 2022. For patients with multiple registered diagnoses, all diagnoses recorded in the Swedish Cancer Registry from January 1, 1958, to December 31, 2022, were retrieved

The NoRCa database consists of data retrieved from registers held at the National Board of Health and Welfare [Socialstyrelsen (NBHW); www.socialstyrelsen.se] and Statistics Sweden [Statistiska Centralbyrån (SCB); www.scb.se]. The baseline population consists of all patients with a registered first cancer diagnosis from 1 January 2007 to 31 December 2022. All diagnoses were linked to a number of healthcare and demographic registries at the NBHW and SCB using a unique Swedish personal identification number (PIN) (see Fig. 2).

### Data retrieved from the national board of health and welfare

#### Swedish Cancer Register (SCR)

Nationwide cancer incidence data have been collected in Sweden since 1958 through the Swedish Cancer Register (SCR) [27]. Reporting of all newly diagnosed malignant tumours is mandatory for physicians and pathologists and is based on clinical and/or histopathological assessment. Diagnoses are morphologically verified in approximately 99% of cases. The SCR includes information on diagnosis codes from different ICD classification systems: ICD-7 (from 1958), ICD-9 (from 1987), ICD-O-2 (from 1993), and ICD-O-3 (from 2005), as well as diagnosis basis, TNM stage (if applicable), FIGO stage for gynaecological cancers, SNOMED morphology codes, and autopsy status. A published quality study estimated the overall completeness at 96% [28].

In addition to the index diagnosis within the time period 2007–2022, information on any multiple tumours was also obtained from the Cancer Register, including diagnoses made prior to 2007.

#### Cause of Death Register (CDR)

The Swedish Cause of Death Register (CDR) [29] is a nationwide, high-quality register that has recorded virtually all deaths in Sweden since 1952. Maintained by the NBHW, the register has been updated annually since 1961. It includes all deceased individuals who were permanent residents of Sweden, regardless of whether they died in Sweden or abroad. The CDR contains information on the date of death, the underlying cause of death, contributing causes, and whether an autopsy was performed. A specific cause of death is recorded for approximately 96% of registered individuals. The overall agreement between register data and medical records has been reported to be 77% and 90% for malignant neoplasms [30].

#### National Patient Register (NPR)

The National Board of Health and Welfare initiated the collection of data in 1964 on patients admitted to public hospitals, which led to the establishment of the National Patient Register (NPR) [31]. Nationwide coverage was achieved in 1987 including information on primary discharge diagnosis, secondary diagnoses, surgical procedures, diagnostic-related group (DRG), hospital and department identifiers, dates of admission and discharge, as well as demographic variables such as sex, age, and place of residence. A diagnostic-related group (DRG) is based on primary and secondary diagnoses, other conditions (comorbidities), age, sex, and necessary medical procedures. Between 1997 and 2000, only day surgery was included from specialized outpatient care. Since 2001, the NPR has also incorporated data on outpatient hospital care—including ambulatory surgical procedures and psychiatric services—from both public and private healthcare providers. Reporting to the register is mandatory for all physicians, and the National Board of Health and Welfare estimates that more than 99% of all hospital admissions are captured [32]. Data from primary care visits are not currently included in the NPR.

Information on diagnoses from the National Inpatient Register and the Outpatient Register is available based on ICD-7 to ICD-10 codes, enabling the calculation of the Charlson Comorbidity Index (CCI) adapted for Swedish registers [33]. The CCI assigns weights to a set of predefined diseases and sums them into a total score, categorized as 0 (none), 1 (mild), 2 (moderate), and ≥ 3 (severe comorbidity).

For patients included in NoRCa, data are available from outpatient care from 1997 and from inpatient care from 1964.

#### Prescribed Drug Register (PDR)

Since July 2005, the Prescribed Drug Register (PDR) [34] has recorded all prescribed medications that are dispensed at pharmacies in Sweden. The register includes information on substance, brand name, formulation, package, date of prescription and dispensing, dispensed quantity, dosage, defined daily doses, prescribing unit (primary care or hospital), and prescriber’s speciality. Medications obtained over the counter without a prescription, as well as drugs administered during hospital care, are not captured in the register.

For patients included in NoRCa, data from PDR are available from 2005.

### Data retrieved from statistics Sweden

#### The Total Population Register (TPR)

The Total Population Register (TPR) [35] contains information from the Swedish Tax Agency about the registered population in Sweden. The TPR was established in 1968, and functions as the basis for official population statistics in Sweden, providing extensive demographic information such as PIN, age, sex, births, deaths, citizenship, civil status, and dates of migration or emigration.

#### Longitudinal integration database for health insurance and labour market studies (LISA)

The LISA database (Longitudinal Integration Database for Health Insurance and Labour Market Studies) [36], established in 1990, comprises all individuals aged 16 years (15 from 2010) and older who were registered as residents of Sweden on 31 December of each year. It consolidates annually updated information from the labour market, educational, and social registers. In the LISA database, the individual is the central unit of analysis, with linkages to family members, employers, and workplaces also available. The database includes variables such as income, country of birth, year of immigration, region of residence, place of employment, highest attained level of education, household size, and socioeconomic status.

A patient’s circumstances, such as employment and income, may be affected by the disease. To also account for a patient’s circumstances up to two years prior to diagnosis, data from LISA are available from 2005.

#### Geographical units

Classification of whether an individual lives in an urban area (at least 200 inhabitants) or a small locality (50 to 199 inhabitants). Urban areas are further classified as either a central urban area (i.e. municipal seat) or other urban area. There are several versions of the boundaries for urban areas and small localities: Urban Areas 2005, 2010, 2015, 2018, 2020; Small Localities 2005, 2010, 2015, 2020.

Demographic Statistical Areas (DeSO) and Regional Statistical Areas (RegSO) [37] are two geographic divisions used for analysing statistics in small areas. DeSO divides Sweden into approximately 6,100 areas, where the population in most cases ranges between 700 and 3,000. The division takes geographical conditions into account, so that, as far as possible, the boundaries follow features such as streets, waterways, and railways. The main components used to construct the first version of DeSO in 2018 were urban areas and electoral districts. DeSO and RegSO are designed to be stable and durable over time, making them suitable for time‑series comparisons.

RegSO is an aggregation of DeSO. RegSO divides Sweden into roughly 3,300 areas, with populations ranging from about 650 to 23,000. If any changes occur in DeSO, the boundaries of RegSO will be adjusted accordingly.

Information on the patients’ location is available for the index year of the date of diagnosis.

#### Distance to nearest hospital and to nearest care unit

For the distance to the nearest hospital or healthcare unit, the residential coordinates are based on the following: 2005–2017: the property point; 2018–2023: the address point, and then the property point if address information is missing. The distance refers to the residence at the time of diagnosis.

The distance is the straight‑line distance between the residence and the healthcare unit, measured in meters and rounded to the nearest whole number.

There are cases where a primary care centre is located inside a hospital and therefore shares the same address points. There are also cases where an individual does not live in the NHCR in a given year; in such cases, they have been excluded from the database.

## Results

As shown in Table 1, a total of 99,522 patients across all ages in the NHCR of Sweden were registered in the SCR with at least one cancer diagnosis between 2007-01-01 and 2022-12-31, with registry data available up to 2023 (a total of 137,074 cancer diagnoses) and were thus eligible for inclusion in NoRCa. Of these, 10,574 individuals had an initial cancer diagnosis prior to 2007 and 152 individuals did not live in the NHCR and were thus excluded, resulting in 88,796 incident cancer cases, 62,340 (70.2%) of whom had recorded TNM or FIGO stage data. A total of 934,196 inpatient and 3,698,679 specialist outpatient care visits were registered.

**Table 1:** Number of non-missing register entries per source/type of data

Variable	*N*	*N* cancer diagnoses since 2007	Unique patients, *N* (%)	Unique patients with first cancer diagnosis since 2007, *N* (%), living in the Northern Healthcare Region
Cancer register	137,074	109,384	99,522	88,796
Outpatient visits	3,698,679	3,199,187	99,362 (99.8)	88,641 (99.8)
Inpatient visits	934,196	793,109	96,556 (97.0)	85,882 (96.7)
Prescribed drugs	34,702,852	29,990,301	99,420 (99.9)	88,697 (99.9)
Deaths			42,860 (43.1)	36,405 (41.0)
Education			98,843 (99.3)	88,144 (99.3)
Income			99,302 (99.8)	88,576 (99.8)
Distance			99,501 (100.0)	88,775 (100.0)

The median age at cancer diagnosis was 68 years (Table 2), 52% of the population were women, and 455 individuals were younger than 18 years old at diagnosis. The most common tumour types in the population were prostate, gynaecological, and breast tumours.

**Table 2:** Descriptive statistics for the five major types of cancer, including non-invasive tumours, and for all types of cancer in the study population

	Breast	Colorectal	Gynaecological	Lung	Prostate	Total
*N*	9554	9541	13,505	4229	15,055	88,796
Any TNM/FIGO record (%)	9023 (94.4)	8554 (89.7)	4777 (35.4)	4067 (96.2)	14,776 (98.1)	62,340 (70.2)
Sex: N (%)						
Male	63 (0.7)	5268 (55.2)	2 (0.0)	2104 (49.8)	15,055 (100.0)	42,522 (47.9)
Female	9491 (99.3)	4273 (44.8)	13,503 (100.0)	2125 (50.2)	0 (0.0)	46,274 (52.1)
Age, median	64	72	37	71	70	68
Age group: N (%)						
0–17		14 (0.1)	15 (0.1)	1 (0.0)		455 (0.5)
18–44	854 (8.9)	289 (3.0)	8417 (62.3)	47 (1.1)	26 (0.2)	12,548 (14.1)
45–54	1706 (17.9)	636 (6.7)	1447 (10.7)	166 (3.9)	531 (3.5)	7657 (8.6)
55–64	2420 (25.3)	1726 (18.1)	1283 (9.5)	772 (18.3)	3522 (23.4)	16,125 (18.2)
65–74	2810 (29.4)	3141 (32.9)	1219 (9.0)	1779 (42.1)	6644 (44.1)	26,349 (29.7)
75–84	1198 (12.5)	2943 (30.8)	871 (6.4)	1260 (29.8)	3678 (24.4)	19,505 (22.0)
85-	566 (5.9)	792 (8.3)	253 (1.9)	204 (4.8)	654 (4.3)	6157 (6.9)

**Fig. 3:**
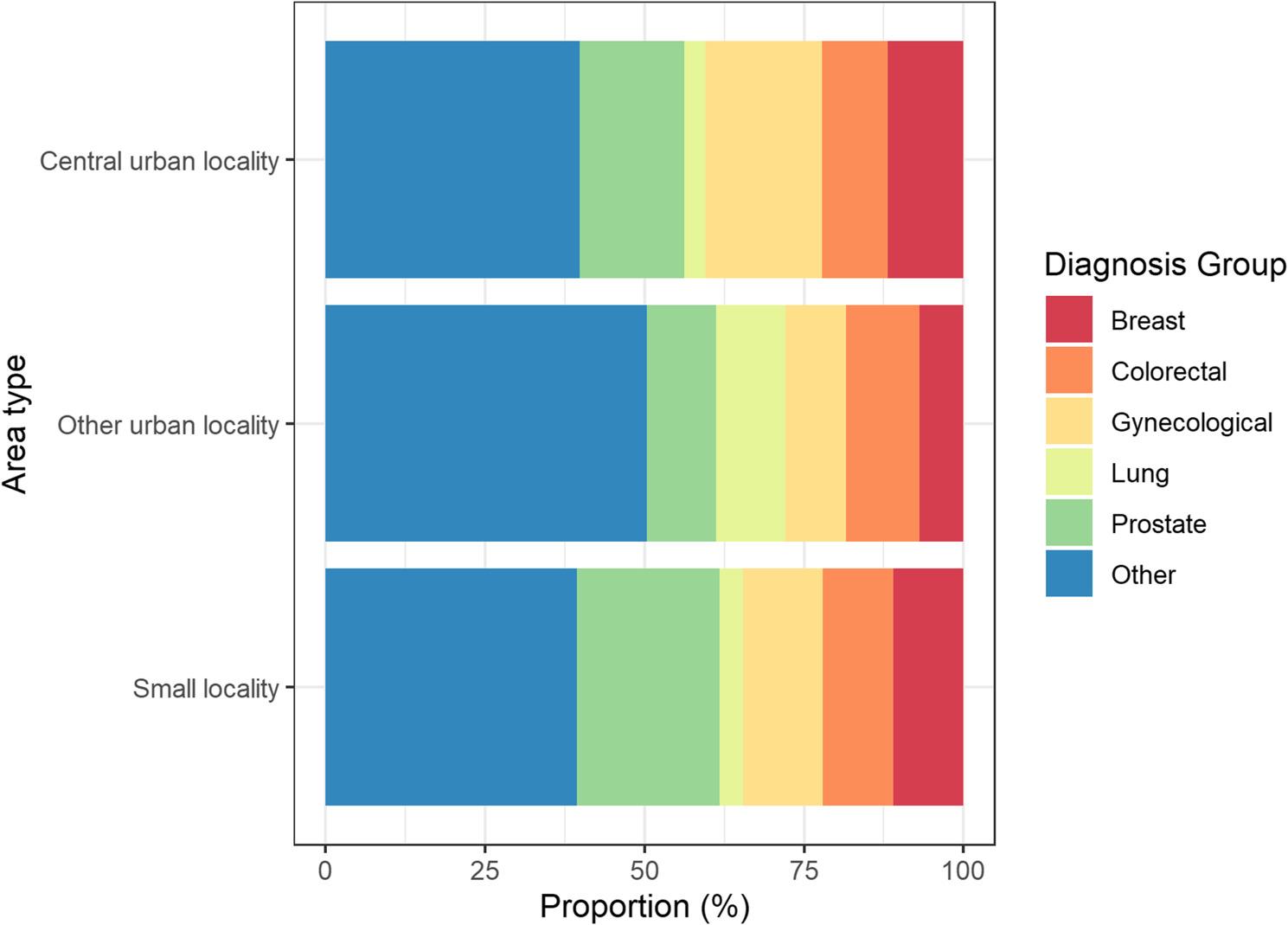
Proportion of the population living in each area type by diagnosis

The most common type of cancer was prostate cancer (Table 2). There was a higher proportion of prostate cancer cases (as an individual’s first cancer diagnosis) in small localities than in central urban or other urban localities (Fig. 3). In central urban localities there was a higher proportion of gynaecological tumours, perhaps due to a higher propensity to attend screening programmes (78% of gynaecological tumours were non-invasive in central urban localities, the corresponding proportions for other urban localities and small localities were 63% and 69%).

## Discussion

In order to examine characteristics among patients living in the NHCR who have been diagnosed with advanced-stage cancer, we developed the comprehensive database NoRCa. This database comprises routinely collected, population-based healthcare data for all individuals diagnosed with cancer in the NHCR of Sweden between 2007 and 2022. The NoRCa database enables examination of geographical, social, healthcare-related, and patient-level characteristics associated with cancer diagnosis. These comprise healthcare-related and patient-level characteristics such as diabetes, comorbidity index, prior medication use, and patterns of healthcare use prior to diagnosis.

In future studies, we aim to examine whether patient and geographical characteristics, particularly distance to care, are associated with advanced-stage cancer diagnosis in the NHCR, using data from the NoRCa database. We will also assess the extent to which socioeconomic factors are associated with tumour stage at diagnosis, drawing on the same data source. The results of such analyses can serve as an important foundation for developing structured approaches and targeted interventions to promote earlier cancer detection in rural areas, in general and in northern Sweden in particular. Ultimately, such efforts have the potential to reduce healthcare inequities and contribute to a more equitable healthcare system. In addition, the integration of data across several registers enables studies addressing a broad range of specific research questions. Overall, NoRCa provides a unique platform for investigating cancer care pathways, with strong potential to inform evidence-based strategies for more equitable and timely cancer care.

### Strengths and limitations

There are several strengths of the NoRCa database that merit comments. First, the high coverage and population‑based design provide a solid foundation for robust analyses, increasing statistical power and improving the validity of the results. Second, the use of personal identity numbers (PINs) enables reliable linkage and integration across several high‑quality national and regional registers. Third, the NoRCa database covers a large geographical area and comprises rich data from multiple registers. This makes the database unique within the northern region, offering valuable information across geographical, healthcare‑related, and individual levels.

A further strength is the inclusion of cause‑of‑death data, enabling analyses of whether mortality patterns differ, for example, between urban and rural areas. The large number of recorded deaths and the reliable cause‑of‑death classification strengthen the validity of the mortality analyses. Similarly, the 15-year study period is a major methodological strength, providing a substantial study population and enabling robust analyses over time.

However, the length of the study period may also pose limitations, as organizational and structural changes may have influenced healthcare provision. Another limitation is the varying quality of information on stage at diagnosis in the cancer registry, which differs across cancer types. Survival time is therefore used as a proxy for late diagnosis.

Although data on time-to-death are generally of high quality, this measure is less informative for diagnoses with low mortality. Additional limitations include the absence of primary care register data, limiting information on pre‑diagnostic contacts and symptom presentations in primary care. Lastly, the SCR only contains data on the diagnosis itself and does not include information on subsequent care, which represents a further limitation.

## Conclusions

The NoRCa database is a unique register-based data source which has the potential to be used in numerous epidemiological studies, particularly for research in sparsely populated regions such as northern Sweden, offering several possible research directions. Moreover, the insights generated through our research agenda can directly inform improvements in the healthcare system, supporting earlier detection efforts and promoting better health outcomes for the population in the northern region.

## Data Availability

The data that support the findings of this study are available from the Swedish National Board of Health and Welfare (Socialstyrelsen) and/or Statistics Sweden (SCB), but restrictions apply to the availability of these data. The data were used under permission for the current study and are therefore not publicly available. Data are however available from the authors upon reasonable request and with permission from the relevant Swedish authorities and following approval in accordance with Swedish legislation.

## Abbreviations

NoRCa: The Northern Region Cancer database
NHCR: Northern Healthcare Region
NBHW: National Board of Health and Welfare
SCB: Statistics Sweden
PIN: Personal identification number
SCR: Swedish Cancer Register
CDR: Swedish Cause of Death Register
NPR: National Patient Register
PDR: Prescribed Drug Register
TPR: Total Population Register
DeSO: Demographic Statistical Areas
RegSO: Regional Statistical Areas
